# Nucleus-specific linker histones Hho1 and Mlh1 form distinct protein interactions during growth, starvation and development in *Tetrahymena thermophila*

**DOI:** 10.1038/s41598-019-56867-0

**Published:** 2020-01-13

**Authors:** Syed Nabeel-Shah, Kanwal Ashraf, Alejandro Saettone, Jyoti Garg, Joanna Derynck, Jean-Philippe Lambert, Ronald E. Pearlman, Jeffrey Fillingham

**Affiliations:** 1 Department of Chemistry and Biology, Ryerson University, 350 Victoria St., Toronto, M5B 2K3 Canada; 2 Department of Biology, York University, 4700 Keele St., Toronto, M3J 1P3 Canada; 3 Department of Molecular Medicine and Cancer Research Centre, Université Laval, Quebec, Canada; 4 CHU de Québec Research Center, CHUL, 2705 Laurier Boulevard, Quebec, G1V 4G2 Canada; 5 Present Address: Donnelly Centre, University of Toronto, Toronto, M5S 3E1 Canada; 6 Department of Molecular Genetics, University of Toronto, Toronto, M5S 1A8 Canada

**Keywords:** Chromatin, Epigenetics, Post-translational modifications, Proteomics

## Abstract

Chromatin organization influences most aspects of gene expression regulation. The linker histone H1, along with the core histones, is a key component of eukaryotic chromatin. Despite its critical roles in chromatin structure and function and gene regulation, studies regarding the H1 protein-protein interaction networks, particularly outside of Opisthokonts, are limited. The nuclear dimorphic ciliate protozoan *Tetrahymena thermophila* encodes two distinct nucleus-specific linker histones, macronuclear Hho1 and micronuclear Mlh1. We used a comparative proteomics approach to identify the Hho1 and Mlh1 protein-protein interaction networks in *Tetrahymena* during growth, starvation, and sexual development. Affinity purification followed by mass spectrometry analysis of the Hho1 and Mlh1 proteins revealed a non-overlapping set of co-purifying proteins suggesting that *Tetrahymena* nucleus-specific linker histones are subject to distinct regulatory pathways. Furthermore, we found that linker histones interact with distinct proteins under the different stages of the *Tetrahymena* life cycle. Hho1 and Mlh1 co-purified with several *Tetrahymena*-specific as well as conserved interacting partners involved in chromatin structure and function and other important cellular pathways. Our results suggest that nucleus-specific linker histones might be subject to nucleus-specific regulatory pathways and are dynamically regulated under different stages of the *Tetrahymena* life cycle.

## Introduction

The fundamental repeating unit of eukaryotic chromatin, the core nucleosome particle, is comprised of two of each of the core histones H2A, H2B, H3 and H4, and ~147 bp of DNA^1^. In addition to the core histones, there are linker histones (H1 or H5) which flank the core nucleosomes sealing the structure with ~20 bp of additional DNA^2^. Within the past three decades, core histones have been extensively studied^3^, however, much less is known regarding the linker histones.

Different numbers of H1 variants have been identified across organisms^4,5^. For example, mammals contain 11 linker histone variants^6^ whereas *Xenopus laevis* and *Caenorhabditis elegans* have five and eight variants, respectively^7^. Most H1 variants are similar in their architecture such that they have an N-terminal region, a C-terminal tail, and a central conserved globular region^2^. *Saccharomyces cerevisiae* linker histone Hho1p is more divergent than its mammalian counterparts and contains two regions of sequence similarity to the central globular domain of the canonical histone H1^8^. Histone H1 has been reported to function in stabilization of chromatin structure^2^, DNA replication^9,10^ as well as gene expression regulation^11^.

Linker histone H1 is known to carry posttranslational modifications (PTMs)^11^. Although the function of H1 PTMs is not well studied, several PTMs including phosphorylation, methylation, acetylation, citrullination, ubiquitylation, formylation, denitration, ADP-ribosylation, crotonylation, and lysine 2-hydroxyisobutyrylation have been identified^2^. Many of the enzymes regulating H1 PTMs are not known^2,11^. Eukaryotic gene expression is subjected to an ever-increasing list of regulatory layers^3^. Recently, metabolic enzymes have been recognized as regulators of various chromatin- and gene expression-related pathways^12^. Various metabolic enzymes, including glycolytic enzymes such as pyruvate kinase M2 isoform (PKM2), 6-phosphofructo-2-kinase/fructose-2,6-bisphosphatase 4 (PFKFB4), fructose-1,6-bisphosphatase 1 (FBP1), glyceraldehyde-3-phosphate dehydrogenase (GAPDH), and tricarboxylic acid (TCA) cycle enzymes such as α-ketoglutarate dehydrogenase (α-KGDH) and fumarase as well as enzymes involved in nucleotide synthesis such as inosine 5′-monophosphate dehydrogenase (IMPDH) and GMP synthase (GMPS), have been shown to localize to the nucleus where they may participate in chromatin regulation by modifying the histones and/or supplying metabolites necessary for histone or chromatin modifying enzymes^12^. For example, PKM2, which functions in glycolysis, was recently shown to directly bind with H3 to mediate phosphorylation at the threonine 11 residue^13^. Whether the role of metabolic enzymes in chromatin regulation is conserved across eukaryotes remains unclear.

The complexes depositing core histones and their variants on chromatin have been extensively studied^3,14^. For example, core histone H3.1 (or H3.2) is assembled onto chromatin only during S phase in a DNA replication dependent manner (RD) by a heterotrimeric CAF1 complex, whereas the variant H3.3 is deposited throughout the cell cycle in a replication independent (RI) fashion by the HIRA histone chaperone^14–17^. Furthermore, many of the generalized H3/H4-specific histone chaperones, such as Asf1 and nuclear autoantigenic sperm protein (NASP), have been identified and extensively studied^18^. Like H3, the chromatin assembly of H2A and its variant H2A.Z (Htz1 in yeast) is also tightly regulated in a cell cycle dependent manner via specialized chaperoning networks^19^. In contrast to the core histones, the deposition complexes of linker histones are not well characterized. Recently, it was reported that human linker histones associated with functionally diverse proteins including RNA-binding proteins, transcriptional regulators, as well as ribosomal proteins^20^. In addition to their roles in core histone metabolism, several proteins including nucleosome assembly protein 1 (Nap1) and NASP, are thought to function as H1 chaperones^2,19,21–23^, although mechanistic details remain largely unknown^24^.

*Tetrahymena thermophila*, a ciliate protozoan, is a well-suited model system to study chromatin biology and investigate gene expression regulatory layers^25–27^. *Tetrahymena* features two distinct nuclei, a macronucleus (MAC) and a micronucleus (MIC), present within the same cell. The polyploid MAC essentially controls all the transcription and divides amitotically during vegetative growth^28^. The diploid MIC is mostly transcriptionally silent, ensures stable inheritance of the genetic material and divides mitotically in vegetatively growing *Tetrahymena*. The two nuclei originate from the same zygotic nucleus during *Tetrahymena* sexual development (conjugation)^28^. During *Tetrahymena* conjugation, extensive chromatin alterations take place in the developing nuclei including DNA rearrangements and removal of ‘internally eliminated sequences (IES)’ giving rise to progeny nuclei with distinct chromatin states^29–31^. *Tetrahymena* conjugation can be induced by starving the cells and mixing cells of two different mating types. Starvation in *Tetrahymena* is a physiological state that is known to induce numerous behavioral, phenotypic and molecular alterations making cells competent to embark on sexual development^32,33^.

The *Tetrahymena* genome encodes two linker histones with distinct nuclear localization^34^. The MAC-specific linker histone Hho1 is thought to be a homolog of mammalian H1 but it lacks the conserved central globular domain^35,36^. Hho1 in *Tetrahymena* in not essential for cell viability and/or chromatin packaging^34^, however, it can regulate expression of certain genes by its differential phosphorylation^37–39^. *Tetrahymena* cells lacking the *HHO1* gene appear to have enlarged MACs but normal MICs^34^. The MIC-specific linker histone 1 (Mlh1) is a much larger protein which is proteolytically processed into smaller fragments (α, β, γ, and δ) after its transport into the MIC^40,41^. The α fragment represents the un-cleaved state of δ and γ fragments. The β and γ fragments appear to resemble H1 without a globular domain whereas the δ fragments have two high mobility group (HMG) boxes. Like Hho1, Mlh1 is not essential for cell viability during vegetative growth. *MLH1* knockout *Tetrahymena* cells display enlarged MICs but normal MACs^34^. The regulatory pathways ensuring the differential transport of nucleus-specific linker histones in *Tetrahymena* remain unknown. Recently, nucleus-specific localization signals (NLS) have been identified for Hho1 and Mlh1^41^. However, the protein factors responsible for the transport of linker histones to specific nuclei have not yet been identified.

Here we employed a comparative proteomic approach to identify the protein-protein interactions of Hho1 and Mlh1 under three different physiological conditions; growth, starvation and conjugation. We carried out affinity purification combined with mass spectrometry (AP-MS) analysis of endogenously tagged Hho1-FZZ and Mlh1-FZZ proteins. Our results reveal that Hho1-FZZ and Mlh1-FZZ interact with distinct sets of proteins under different physiological conditions. Furthermore, we found that the *Tetrahymena* linker histones interact with non-overlapping proteins suggesting that the nucleus-specific linker histones have distinct regulatory pathways. We suggest that *Tetrahymena* linker histones are dynamically regulated under distinct physiological stages to ensure their faithful transport and deposition to the specific nuclei.

## Results

### Engineering endogenously tagged Hho1-FZZ cell lines

**Figure 1:**
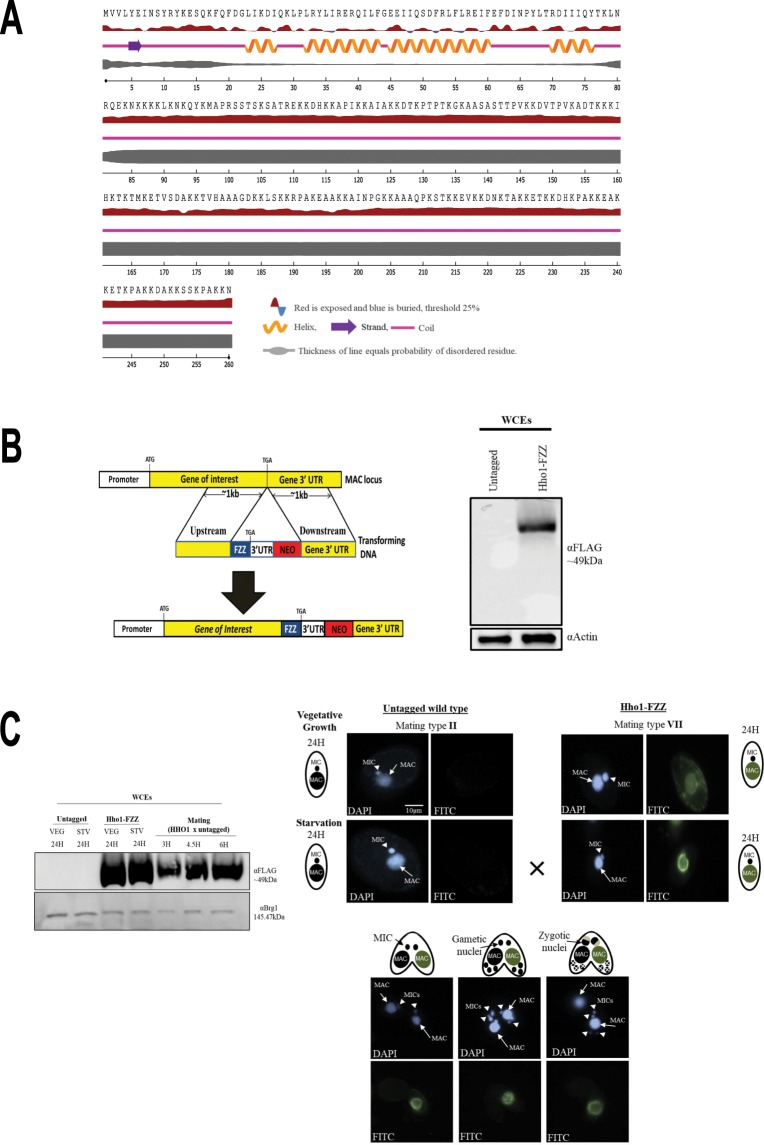
Hho1-FZZ expression and localization in the MAC. (**A**) Predicted secondary structure of *Tetrahymena* MAC-specific linker histone Hho1. Hho1 protein is mostly composed of disordered regions. (**B**) Left- Schematic representation of epitope tagging strategy for the MAC locus. Right- Expression analysis of endogenously tagged Hho1-FZZ in comparison to the untagged controls by Western blotting in whole cell extracts (WCEs). Top panel was probed with anti-FLAG in order to detect Hho1-FZZ and the bottom panel was probed with anti-Actin as a loading control. **(C**) Left- Western blotting analysis using Hho1-FZZ WCEs to examine its expression across the indicated stages of the *Tetrahymena* life cycle. VEG-vegetative growth; STV-starvation; mating-conjugating *Tetrahymena* cells. Top panel was probed with anti-FLAG antibody to detect Hho1-FZZ and the bottom panel was probed with anti-Brg1 as a loading control. Right- HHo1-FZZ localizes to the MAC only and not to the MIC during vegetative growth, starvation and conjugation. Hho1-FZZ cells of mating type VII were mated with the untagged cells of mating type II. Nuclear events are depicted as cartoons. DAPI was used to stain the nuclei. The images were processed using ImageJ (version 1.50i) (https://imagej.nih.gov/ij/).

The *Tetrahymena* genome contains a single gene encoding MAC-specific linker histone *HHO1*, the protein product of which shares similarity with the C-terminal region of the metazoan H1 but lacks the central globular domain^35,36^. Our secondary structure prediction of Hho1 indicated that its N-terminus (residues 1–75) is composed mostly of helices whereas the remainder of the protein is largely disordered, consistent with the structure of the metazoan linker histone C-terminus^42^ (Fig. 1A).

Toward our goal of identifying Hho1 protein-protein interactions, we generated a *Tetrahymena* cell line stably expressing *HHO1* with a C-terminal FZZ epitope tag from its endogenous MAC locus (Fig. 1B, left). We previously successfully employed the FZZ epitope tag, comprised of 3 × FLAG and 2 protein A moieties separated by a TEV cleavage site, to perform affinity purification experiments and conduct indirect immunofluorescence (IF) studies^25^. The expression of endogenously tagged *HHO1-FZZ* was confirmed by Western blotting analysis in whole cell extracts (WCEs) prepared either from *HHO1-FZZ* expressing cells or untagged wild-type *Tetrahymena* (Fig. 1B, right). We next examined the expression levels of Hho1-FZZ during vegetative growth, starvation and conjugation 3-, 4.5- and 6-hours post mixing the different mating types. We observed that Hho1-FZZ is abundantly expressed throughout the examined stages of the *Tetrahymena* life cycle (Fig. 1C, left). To further examine the functionality of endogenously tagged Hho1-FZZ, we carried out IF analysis through the above stages of the *Tetrahymena* life cycle. Consistent with previous studies^41^, we found that Hho1-FZZ is exclusively localized to the MAC throughout the examined stages of the *Tetrahymena* life cycle (Fig. 1C, right), indicating that the FZZ tag does not interfere with the proper localization of Hho1-FZZ.

### Interactome mapping of Hho1 throughout *Tetrahymena* life cycle

**Figure 2:**
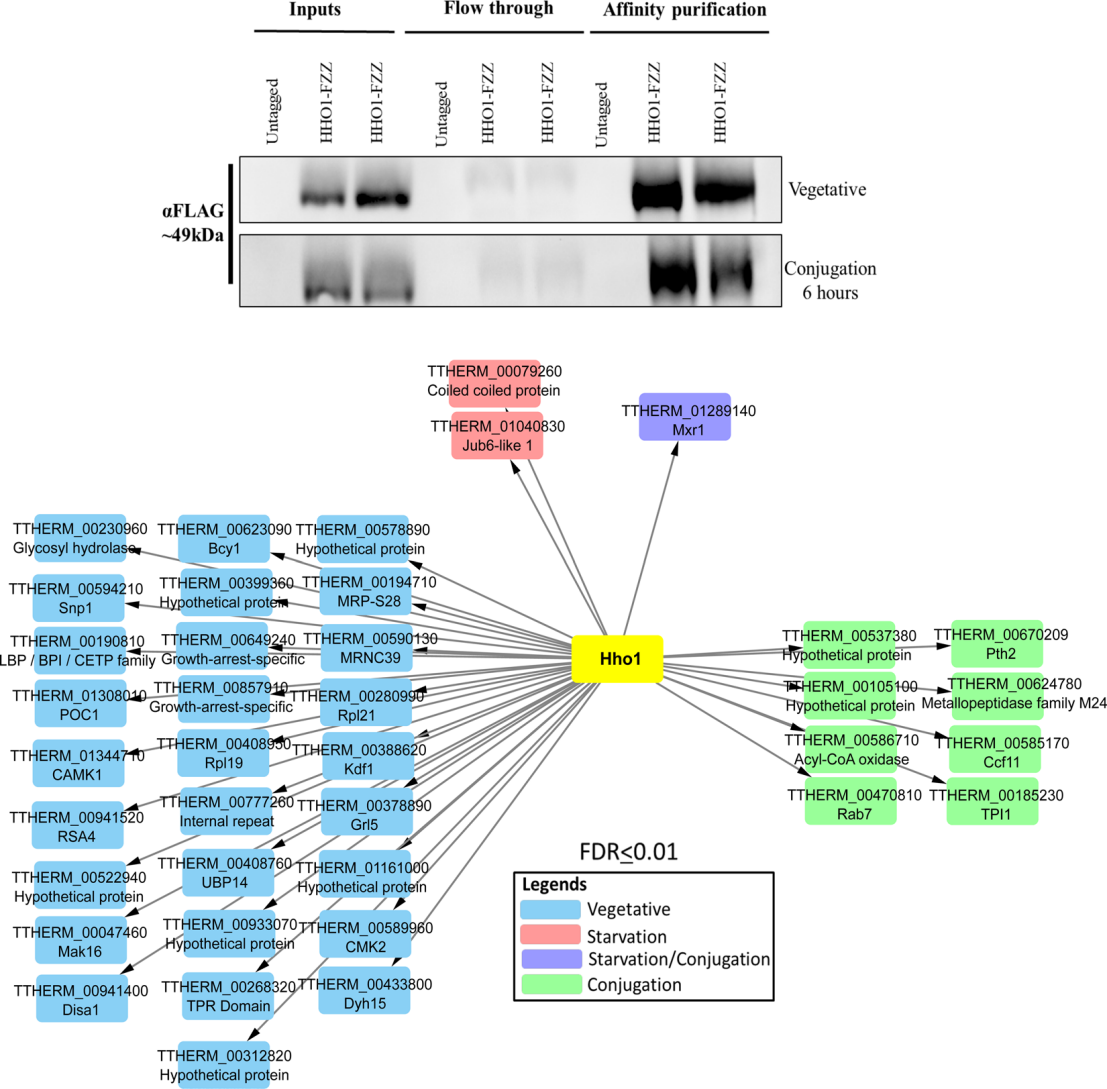
AP-MS analysis of Hho1-FZZ in three different physiological states of the *Tetrahymena* life cycle. Top: Western blotting analysis indicating recovery of the affinity purified Hho1-FZZ bait protein. The top panel represents the vegetative samples and the bottom panel shows the recovery of the bait from conjugation samples. The blots were probed with anti-FLAG antibody. No signal was detected in samples from the untagged control cells. Bottom: Network illustration of the Hho1-FZZ co-purifying proteins at statistical threshold of FDR ≤ 1% in three different stages of the *Tetrahymena* life cycle; vegetative growth, starvation (24 hours) and conjugation (6 hours post mixing of the cells). Node color key is provided.

To identify Hho1-FZZ protein-protein interactions (PPIs), we performed affinity purification (AP) coupled to mass spectrometry analysis (AP-MS). We adopted a comparative proteomics approach and examined Hho1 interaction profiles through different stages of the *Tetrahymena* life cycle including growth, starvation (24 hours) and development (6 hours post mixing, a time when most mating cells have finished post-zygotic nuclear divisions). The recovery of affinity purified bait was monitored by Western blotting (Fig. 2, top). We used ‘Significance Analysis of INTeractome’ (SAINTexpress) analysis to evaluate our MS data^43^. The SAINTexpress utilizes semiquantitative spectral counts for assigning a confidence value to individual PPIs^43^. We used untagged wildtype *Tetrahymena* cells for control purifications from the appropriate cell cycle stages (i.e., during growth, starvation or development). The Hho1-FZZ AP-MS data filtered against control purifications identified a set of interacting partners that passed the statistical threshold (Bayesian false discovery rate (FDR) ≤ 1%) and are depicted in Fig. 2 (Supplemental File S1).

Hho1-FZZ co-purified with distinct interaction partners through the examined stages of the *Tetrahymena* life cycle (Fig. 2, bottom). The Hho1-FZZ co-purifying proteins were analyzed for domain architecture and possible similarities to budding yeast and/or human proteins. During vegetative growth Hho1-FZZ co-purified with 28 high-confidence (FDR ≤ 1%) interacting partners. Among the vegetative-specific Hho1-FZZ co-purifying proteins were 6 hypothetical proteins without any recognizable domains, two growth-arrest-specific 8 (GAS8) proteins, a tetratricopeptide (TPR) repeat domain protein, two putative protein kinases including Camk1 and Cmk2, ribosomal proteins Rpl19 and Rpl21, Ubp14 and the putative splicing factor Snp1 (U1 small nuclear ribonucleoprotein 70 kDa homolog) (Fig. 2; see Supplemental File S1 for details and the full list of Hho1 PPIs). Additionally, we identified Jub6-like 1 (Jsl1 (TTHERM_01040830)) protein to interact with Hho1 during vegetative growth at a slightly relaxed statistical threshold of BFDR ≤ 0.02. Jsl1 is closely related to a prion-like protein Jub6 in *Tetrahymena* which has previously been shown to be involved in the regulation of chromatin structure^44^.

Application of SAINTexpress revealed three high-confidence Hho1-FZZ interaction partners in starving *Tetrahymena* cells. These include a coiled-coil domain protein, Jsl1, which was also identified in vegetative growth (see above), and Mxr1 (methionine-S-sulfoxide reductase 1). Mxr1 was also identified as a high-confidence Hho1-FZZ interacting partner during conjugation. The SAINTexpress analysis identified eight additional high-confidence Hho1-FZZ co-purifying proteins in conjugating *Tetrahymena* cells including TTHERM_00585170 which encodes a dense core granule associated C-terminal crystallin fold protein Ccf11, and two hypothetical proteins TTHERM_00105100 and TTHERM_00537380 both of which lack recognizable domains and appear to be *Tetrahymena*-specific without identifiable sequence similarity in any other organism. Based on sequence similarity to the budding yeast proteins, the remaining conjugation-specific Hho1-FZZ interaction partners include an acyl-CoA oxidase Pox1, rab-family GTPase Rab7 (or Ypt7), glycolytic enzyme Tpi1, peptidyl-tRNA hydrolase Pth1 and a metallopeptidase family M24 containing protein Fra1. Additionally, Jub6 was identified to co-purify with Hho1-FZZ during conjugation. It however did not pass our stringent statistical threshold of FDR ≤ 0.01.

We used publicly available micro-array data^45^ to examine the expression profiles of Hho1-FZZ co-purifying proteins (Supplementary Fig. S1). We observed that Hho1-FZZ co-purifying partners have expression profiles that often correlated with the physiological state in which the interactions were detected. This suggests that Hho1 and its interacting partners are functionally linked. It is important to note that the expression patterns do not always correlate with the interaction profiles ruling out the possibility of detecting false positive interactions in the AP-MS. For example, several of the Hho1-FZZ interaction partners detected during vegetative growth (e.g. CETP, GAS8 and MRNC9) have the highest expression levels either during starvation or conjugation (Supplementary Fig. S1).

### Structural analysis of Mlh1 and of the high mobility group protein family in *Tetrahymena*

**Figure 3:**
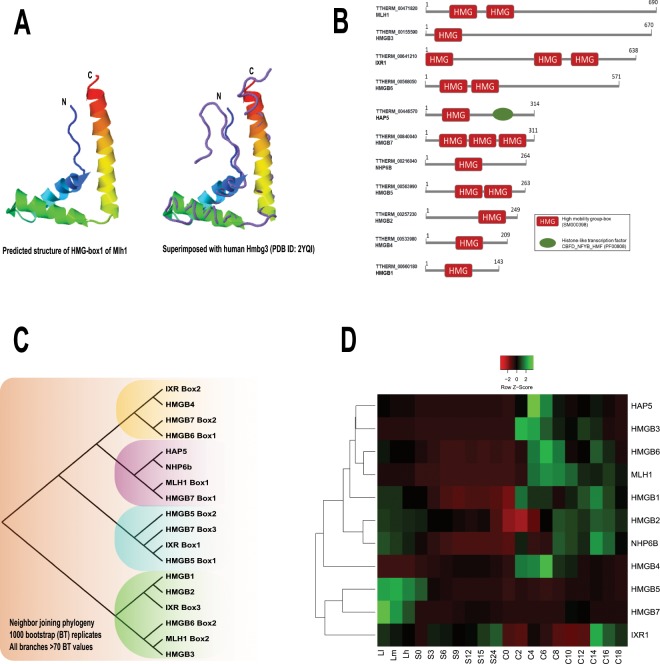
Mlh1 and HMG-box proteins in *Tetrahymena*. (**A**) Cartoon diagram shows the predicted structure of the *Tetrahymena* Mlh1 HMG-box 1 in rainbow color. The N- and C-termini are indicated. **(B**) Domain architecture of the HMG-family proteins identified in *Tetrahymena*. Domain legends are provided in the box. **(C**) Neighbour-joining phylogeny of HMG-box amino acid sequences from the identified HMG-family proteins in *Tetrahymena*. 1000 bootstrap replicates were performed to test the reliability of the resulting tree. All the nodes depicted had >70 bootstrap values. Node length does not indicate the genetic distance. **(D**) Heatmap represents the expression profiles based on hierarchical clustering of the microarray expression values for the HMG-family proteins across different stages of the *Tetrahymena* life cycle. Z-scores were calculated across each row to examine the differential expression across different stages. L1-LH: vegetative growth, S0-24: starvation for 24 hours, and **C**: conjugation (0–18 hours post mixing the cells of different mating types).

The *Tetrahymena* genome encodes a distinct MIC-specific linker histone gene *MLH1*. Mlh1 protein is more than twice the length of Hho1 and contains two high-mobility group (HMG) boxes. The HMG domain is highly conserved throughout the eukaryotes and is known to function as chromatin architectural proteins^46^. Our structural analysis indicated that both HMG boxes in Mlh1 are composed of helices forming an ‘L-shaped’ fold, typical of the HMGs^47^ (Fig. 3A). Previous studies have found that the Mlh1 and another MIC-specific HMG-family protein, HmgB3, have similar expression profiles through *Tetrahymena* developmental stages, and that HmgB3 could functionally compensate for the loss of *MLH1*^48^. Both the Mlh1 and HmgB3, however, when knocked out individually are not essential for *Tetrahymena* growth suggesting that there might be additional functionally redundant proteins^48^. We searched the *Tetrahymena* genome for HMGs and identified at least 11 genes encoding proteins with an HMG-box (Fig. 3B). To infer the genetic relationship and predict any functional similarities that might exist among the identified proteins, we carried out a phylogenetic analysis using the HMG-box amino acid sequences (Fig. 3C). Consistent with their proposed functional link^48^, HmgB3 and HMG-box 2 of Mlh1 clustered together. Another HMG-box protein HmgB6 (HMG-box 2) also clustered with HmgB3 and Mlh1, suggesting a functional link among these proteins. We analyzed the expression profiles of the identified *Tetrahymena* HMG-family proteins using publicly available micro-array data^45^ (Fig. 3D). Our analysis revealed that the expression of HMG-family proteins is temporally regulated during different stages of *Tetrahymena* development. Consistent with our phylogenetic analysis, HmgB6, HmgB3 and Mlh1 clustered together due to their very similar expression profiles (Fig. 3D), supporting the idea that, like HmgB3, HmgB6 might also be functionally redundant with Mlh1.

### Functional proteomic analysis of Mlh1 during growth, starvation and development

**Figure 4:**
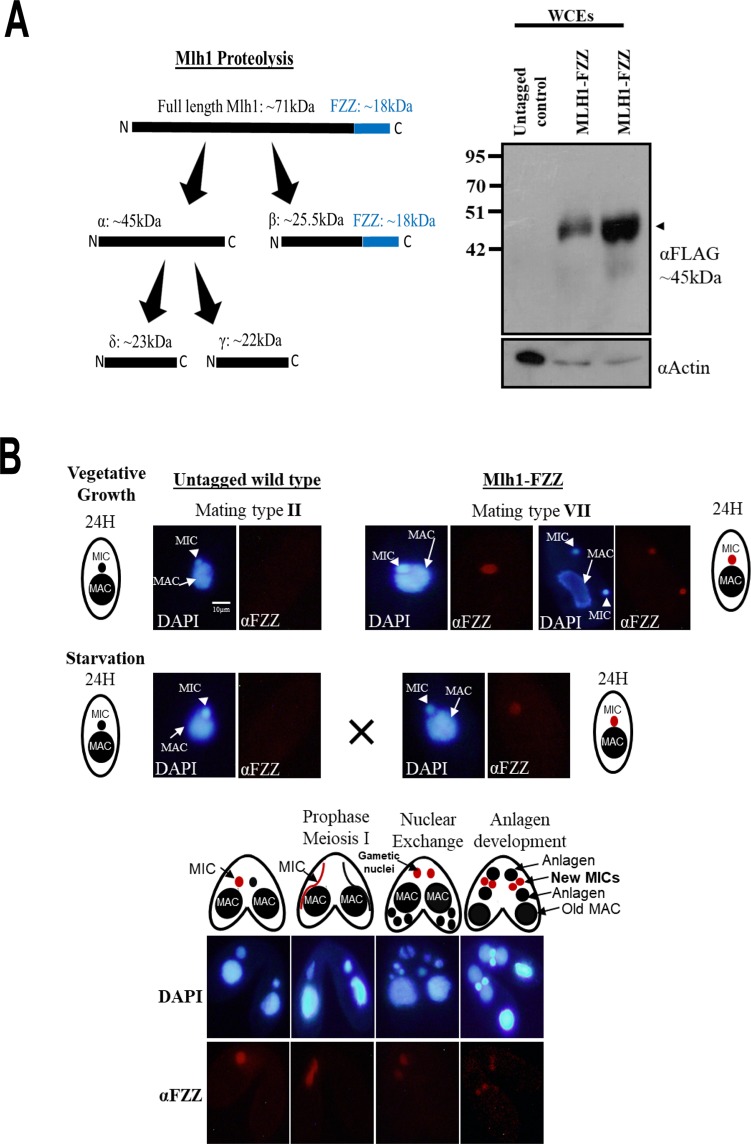
Mlh1-FZZ exclusively localizes to the MIC. (**A**) Left: Schematic representation of proteolytic cleavage of full length Mlh1. The predicted product size and molecular weights are indicated. The position and size of the FZZ tag is indicated in blue. Proteolysis of the full-length Mlh1-FZZ results in β-FZZ product. Right: Expression analysis of endogenously tagged Mlh1-FZZ in comparison to the untagged controls by Western blotting in WCEs. The black arrowhead represents the β fragment (~25.5 kDa + FZZ 18 kDa). Anti-FLAG antibody was used to probe the top panel for Mlh1-FZZ detection. The bottom panel was probed with anti-Actin as a loading control. Two separate clones of Mlh1-FZZ were analysed. **(B**) Mlh1-FZZ localizes to MIC only during vegetative growth, starvation and conjugation. Mlh1-FZZ cells (mating type VII) were mated with untagged cells of mating type II. Nuclear events are illustrated as cartoons. Mlh1-FZZ signal was observed only in the MICs of the FZZ tagged cells and not in the untagged controls. The signal observed in both mating pairs (Mlh1-FZZ and controls) at the anlagen stage indicates mixing of cellular contents between the pairing cells. DAPI was used to stain the nuclei. The images were processed using ImageJ (version 1.50i) (https://imagej.nih.gov/ij/).

We engineered a *Tetrahymena* cell line stably expressing *MLH1* with a C-terminal FZZ epitope tag from its endogenous MAC locus. Western blotting analysis demonstrated the successful expression of endogenously tagged Mlh1-FZZ protein in WCEs prepared from the epitope-tagged strain (Fig. 4A). Consistent with previous studies^40^ (Fig. 4A; left), we found that full-length Mlh1-FZZ is not detectable due to its known proteolysis into four distinct fragments (Fig. 4A). To examine the subcellular localization of Mlh1-FZZ, we carried out IF analysis through different stages of the *Tetrahymena* life cycle including vegetative growth, starvation and sexual development. Consistent with previous studies^41,49^, our IF analysis indicated that Mlh1-FZZ exclusively localized to the MIC during *Tetrahymena* vegetative growth, starvation and conjugation stages examined (Fig. 4B). We also examined the Mlh1-FZZ localization in mitotically dividing MICs during vegetative growth and consistently observed a signal only in the MICs and not in the MACs (Fig. 4B). These results indicate that endogenously tagged Mlh1-FZZ is competent for proteolysis and nuclear import and that the presence of an FZZ tag does not alter its known localization profile.

**Figure 5:**
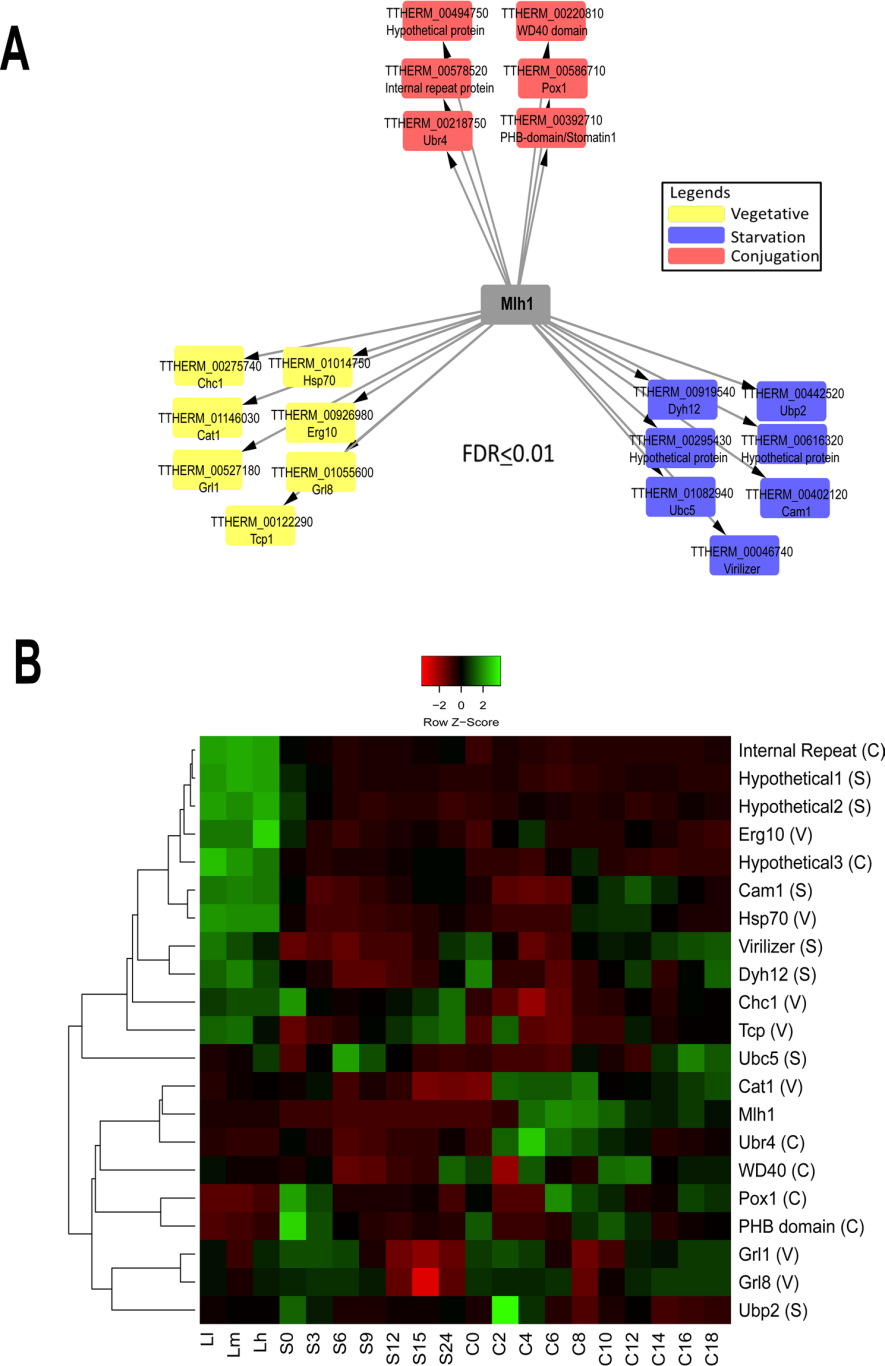
Mlh1-FZZ protein-protein interaction network. **(A**) A Network illustration of the Mlh1-FZZ co-purifying proteins from three different stages of the *Tetrahymena* life cycle (statistical threshold of FDR ≤ 1%). The node color legend is provided. (**B**) Heat map of microarray expression values for Mlh1-FZZ co-purifying proteins in the three different stages of the *Tetrahymena* life cycle. Z-scores were calculated across the rows for each protein to examine its differential expression across growth, starvation and various developmental stages. L1-LH: vegetative growth phase, S0-24: starvation for 24 hours, and C: conjugation where 0–18 denotes hours post mixing the different mating types. V, S and C in parenthesis after each prey protein name indicate vegetative, starvation and conjugation-specific interactions.

To identify Mlh1-FZZ PPIs, we performed AP-MS experiments in biological replicates to examine Mlh1 interaction profiles through different stages of the *Tetrahymena* life cycle, including log-phase growth, starvation (24 hours) and development (6 hours post mixing, a time when most mating cells have finished post-zygotic nuclear divisions). Of note, previous work showed that the full-length Mlh1 is cleaved into four peptides upon its import to the MIC^41^. By WB (Fig. 4A), we found β-FZZ to be the major fragment observed suggesting that our AP-MS analysis captures the PPIs of the β-FZZ fragment. The full-length construct may also contribute to our MLH1 AP-MS results but to a lower degree. We used SAINTexpress analysis to evaluate our MS data^43^. The Mlh1-FZZ AP-MS data in biological replicates filtered against several control purifications yielded a set of 20 high-confidence co-purifying proteins (FDR ≤ 1%) (Fig. 5A; Supplemental File S1) through three distinct *Tetrahymena* physiological conditions.

During vegetative growth, Mlh1-FZZ co-purified with seven high-confidence binding partners including a heat shock protein Hsp70 and TCP-1/cpn60, both of which are thought to have a role in proper folding of other proteins^50,51^ (Fig. 5A). Of note, the heat shock proteins including Hsp70 and Hsp90 have previously been shown to function in proper folding of newly synthesized histones H3/H4^52^. Additionally, SAINTexpress identified two *Tetrahymena* granule lattice proteins including Grl1 and Grl8, a peroxisomal enzyme Cat1, coat protein Chc1 and a putative homolog of budding yeast metabolic enzyme acetyl-CoA acyltransferase Erg10. Application of SAINTexpress to the Mlh1-FZZ AP-MS data from starving *Tetrahymena* cells revealed seven high-confidence interacting proteins including two hypothetical proteins, TTHERM_00295430 and TTHERM_00616320, both of which appear to be ciliate-specific and lack any recognizable domains. The SAINTexpress analysis identified TTHERM_00442520, TTHERM_01082940 and TTHERM_00402120 to copurify with Mlh1-FZZ in starving cells. These proteins share sequence similarities to budding yeast ubiquitin-specific protease Ubp2, ubiquitin-conjugating enzyme Ubc5 and nuclear protein Cam1, respectively. Although, ubiquitination of *Tetrahymena* Mlh1 is yet to be examined, co-purification of ubiquitin ligase suggests a role for this PTM in Mlh1 metabolism. Additionally, THERM_00046740 was identified as a high-confidence starvation-specific Mlh1-FZZ co-purifying protein which shares sequence similarity with human VIRMA (protein Virilizer homolog) protein and contains a signature VIR_N domain (Pfam accession: pf15912) (Supp. Fig. S2). In humans, VIRMA was recently shown to co-purify with linker histones^20^. In *Drosophila melanogaster*, Virilizer has been shown to be crucial for male and female viability and is required to produce eggs capable of embryonic development^53^.

Application of SAINTexpress revealed six high-confidence conjugation-specific (6 hours post mixing the different mating type *Tetrahymena* cells) Mlh1-FZZ interaction partners including hypothetical proteins TTHERM_00494750, TTHERM_00220810 and TTHERM_00578520. TTHERM_00494750 and TTHERM_00220810 contain internal repeats and a WD40 domain, respectively, whereas TTHERM_00578520 does not have any identifiable domains. The WD40 domain is thought to provide a scaffold to mediate protein-protein interactions and is found in many proteins with chromatin-related functions^54^. Additionally, SAINTexpress identified a PHB-domain containing protein TTHERM_00392710, a putative E3 ubiquitin ligase Ubr4 TTHERM_00218750 and a hypothetical protein TTHERM_00586710 which shares similarity to yeast fatty-acyl coenzyme A oxidase POX1 (Fig. 5A). Expression analysis using publicly available microarray data^45^ revealed that many of the conjugation-specific Mlh1-FZZ co-purifying proteins clustered together and their expression is increased between 4–8 hours post-mixing of the cells (Fig. 5B).

## Discussion

Linker histones are a key component of eukaryotic chromatin. Recent studies indicate that linker histone H1 functions in multiple chromatin-related processes including DNA replication^9^, DNA- repair^55^, and modulation of epigenetic information^2^. The information regarding the linker histone chaperones and deposition complexes outside of the Opisthokonts remains limited^11,20^. Here we report the proteomic analysis of *Tetrahymena* nucleus-specific linker histones Hho1 and Mlh1. We utilized a comparative proteomic approach to profile the protein-protein interactions for the MAC-specific Hho1 and MIC-specific Mlh1 through different stages of the *Tetrahymena* life cycle including growth, starvation and conjugation. We found that the nucleus-specific linker histones generally formed distinct non-overlapping protein-protein interactions under different physiological conditions.

We have previously reported the protein-protein interaction networks for the core histones H2A, H2B and variant Hv1 in *Tetrahymena*^25^. The core histones H2A(Hv1)/H2B in *Tetrahymena* were found to interact with a highly conserved network of dedicated chaperones including the FACT-complex and nucleoplasmin1-like (cNPL1) protein^25^. Furthermore, we have previously shown that conserved histone chaperones Asf1 and NASP (Nrp1) likely function in H3/H4 metabolism in *Tetrahymena*^56,57^, consistent with their known roles in other organisms^14^. We observed that the linker histones in *Tetrahymena* co-purify with functionally diverse as well as several lineage-specific proteins that lack orthologs in other organisms. This suggests that *Tetrahymena* linker histones might be subject to lineage-specific regulatory pathways. These observations are consistent with a recent proteomics analysis of human linker histone variants^20^. This study found that functionally diverse proteins including RNA-binding proteins, chromatin-related factors, ubiquitin specific peptidase as well as nucleolar proteins co-purify with linker histones^20^. Among the known human histone chaperones, Nap1L1 is thought to function as a linker histone chaperone in addition to its role as a core histone binding protein^2,58,59^. The *Tetrahymena* genome encodes TTHERM_00786930 which shares high sequence similarity with human and yeast Nap1 proteins. The *Tetrahymena* Nap1 did co-purify with Hho1 in all three physiological conditions that we examined and with Mlh1 only during conjugation. It however did not pass our statistical threshold of FDR ≤ 1%. Identifying the protein interaction network of Nap1 would provide insights into its possible functions in *Tetrahymena*. We suggest that unlike core histones with dedicated chaperoning networks that arose early during eukaryotic evolution^14,25,26^, the linker histones might be more flexible in terms of their interaction partners and may lack a set of conserved specialized chaperones.

Recent evidence suggests that histones are post-translationally modified by various metabolic enzymes, e.g. PKM2, PFKFB4, FBP1, GAPDH and α-KGDH^12^. Furthermore, linker histone H1 is also known to be ubiquitinated^60,61^. Our AP-MS data revealed that several putative metabolic enzymes co-purify with the linker histones (Supplementary Data S1; Figs. 2 and 5). This observation raises the possibility that *Tetrahymena* linker histones might also carry PTMs, such as acylation and ubiquitylation, although such epigenetic marks are yet to be examined in this model organism.

Jub6 protein forms prion-like aggregates and is a component of heterochromatin bodies in the developing MAC during *Tetrahymena* conjugation^44^. The *Tetrahymena* genome appears to encode at least six Jub6-like proteins (Jsl 1–6)^44^. These proteins however do not appear to have prion-like properties and their functions remain unknown^44^. Hho1 in *Tetrahymena* is differentially phosphorylated during vegetative growth and starvation^37–39^. The phosphorylation of Hho1 has been shown to regulate transcription and mimics the loss of *HHO1*^37^. In starving *Tetrahymena*, Hho1 is dephosphorylated which allows it to associate with chromatin and positively or negatively regulate the expression of certain genes^37–39^. Considering that H1 has been shown to be important for heterochromatin formation in other organisms^62^, and starvation in *Tetrahymena* features Hho1 dependent compaction^37–39^, it will be interesting to investigate the interplay between Hho1 and Jsl1.

Human VIRMA protein has recently been shown to co-purify with human linker histone variants^20^. The co-purification of a putative VIRMA ortholog with Mlh1 suggests a conserved role of VIRMA in linker histone metabolism. Recently, VIRMA was shown to function in mediating N6-methyladenosine (m6A) methylation of mRNAs as a component of the WMM complex^63^. In *Drosophila melanogaster*, Virilizer plays a crucial role in male and female viability and is thought to be required to produce eggs capable of embryonic development^53^. Starvation in *Tetrahymena* is known to induce distinct phenotypic alterations and molecular changes resulting in cellular competence for sexual development^32,33^. The co-purification of a putative VIRMA protein with Mlh1 in starving *Tetrahymena* cells suggests a possible role for it in preparing the cellular chromatin for sexual development.

In summary, our study presents the first profile of the *Tetrahymena* linker histone protein-protein interactions. Our results demonstrate that *Tetrahymena* MAC- and MIC-specific linker histones form distinct protein interaction networks under different physiological conditions. Carrying out BioID-MS^64^, an orthogonal approach to AP-MS, will be instrumental to provide a more detailed view of Mlh1 and Hho1 interaction networks.

## Materials and Methods

### Cell strains and culture conditions

*Tetrahymena* inbreeding line B strains CU428 [Mpr/Mpr (VII, mp-s)] and B2086 [Mpr+/Mpr+ (II, mp-s)] were acquired from the *Tetrahymena* Stock Center, Cornell University, Ithaca N.Y. (http://tetrahymena.vet.cornell.edu/). Cells were cultured in 1 × SPP media and were maintained axenically at 30 °C as previously described^65^. To induce conjugation, exponentially growing *Tetrahymena* cells of two different mating types were starved overnight at 30 °C without shaking in 10 mM Tris-HCl pH 7.5. The starved cells were mixed in roughly equal numbers (5 × 10^5^–7 × 10^5^ cells/mL) to allow conjugation at 30 °C without shaking.

### Macronuclear gene replacement

To construct the epitope tagging vectors for *HHO1* and *MLH1*, ~1 kb DNA fragments upstream and downstream of the predicted stop codons of each gene were amplified by standard PCR reactions. The PCR products were digested with KpnI/XhoI and NotI/SacI for the upstream and downstream fragments, respectively. The digested products were cloned into the appropriate sites within the tagging vector (pBKS-FZZ) provided by Dr. Kathleen Collins (University of California, Berkeley, CA). The final plasmid was double digested with KpnI and SacI for linearization prior to biolistic transformation into *Tetrahymena* cells. We used one micrometer gold particles (60 mg/mL; Bio-Rad) and coated them with 5 μg of the linearized plasmid DNA. The gold particles were introduced into the *Tetrahymena* MAC via biolistic transformation with a PDS-1000/He Biolistic particle delivery system (Bio-Rad). The transformants were selected using paromomycin (60 μg/mL). MAC homozygosity was achieved by growing the cells in increasing concentrations of paromomycin to a final concentration of 1 mg/ml.

### Generation of whole cell extracts (WCE) and Western blotting

10% trichloroacetic acid was used to prepare WCE by incubation on ice for 30 minutes. The WCEs were re-suspended in 500 μL of SDS loading dye. 10 μL of 1 N NaOH was added to neutralize the final solution, if required. WCEs were subjected to electrophoresis through 10% SDS-PAGE. Proteins transferred to a nitrocellulose membrane were blocked in 5% skim milk and probed with the indicated antibodies. Antibodies and dilutions used were anti-Flag (1:4000; Sigma), anti-Actin (1:10000; Abcam), anti-Brg1 (1:1000, as described^66^)

### Affinity purification and mass spectrometry sample preparation

Affinity purification was carried out using our previously described method^25,56^. Briefly, *Tetrahymena* were grown in ~500 mL of 1 × SPP to a final concentration of 3 × 10^5^ cells/mL and were pelleted. For starvation, cells were washed once with 1 × PBS, resuspended in 10 mM Tris-HCl pH 7.5 and incubated at 30 °C for 24 hours without shaking before pelleting. To induce conjugation, 24 hours starved cells of different mating types were mixed in equal numbers and incubated at 30 °C for 24 hours without shaking. After 6 hours post mixing, cells were pelleted. The cell pellets for each of the above conditions were frozen at −80 °C until further use. The pellets were thawed on ice and re-suspended in lysis buffer (10 mM Tris–HCl pH 7.5, 1 mM MgCl_2_, 300 mM NaCl and 0.2% NP40 plus yeast protease inhibitors (Sigma)). To eliminate the contaminating chromatin and/or RNA, Benzonase (Sigma E8263) nuclease was added (500 units) and extracts were rotated for 30 minutes at 4 °C. WCEs were clarified by centrifugation at 16,000 g for 30 minutes, and resulting soluble material was incubated with 50 μL of packed M2-agarose (Sigma) at 4 °C for 3–4 hours. The M2-agarose was washed once with 10 mL IPP300 (10 mM Tris–HCl pH 8.0, 300 mM NaCl, 0.1% NP40), two times with 5 mL of IP100 buffer (10 mM Tris–HCl pH 8.0, 100 mM NaCl, 0.1% NP40), and two times with 5 mL of IP100 buffer without detergent (10 mM Tris–HCl pH 8.0, 100 mM NaCl). 500 uL of 0.5 M NH_4_OH was used to elute the proteins by rotating the samples at room temperature for ~20 minutes.

### Experimental design for mass spectrometry experiments

Two biological replicates were employed for each bait under study and processed independently. The samples were analysed alongside negative controls in each batch processed. For negative controls, we used untagged *Tetrahymena* cells (i.e. empty cells). In order to minimize the carry-over, extensive washes were performed between each sample (see details for each instrument type); and the order of sample acquisition on the mass spectrometer was reversed for the second replicate to circumvent systematic bias.

### Mass spectrometry sample preparation

Preparation of protein eluates for mass spectrometry acquisition was essentially as previously described^67^. Briefly, the eluates were dried using a speed vacuum apparatus and re-suspended in 10 μL of 20 mM Tris-HCl pH 8.0. Trypsin digestion was carried out using 0.75 μg of trypsin (Sigma) for ~ 15 hours at 37 °C with mild agitation. An extra 0.25 μg of trypsin was added to each sample and they were incubated for an additional 3 hours. The samples were acidified to a final concentration of 2% acetic acid, desalted using C_18_ StageTips (Thermo Scientific) as per the manufacturer’s instructions and stored at −80 °C until their acquisition on a mass spectrometer.

### Mass spectrometry acquisition using a Triple TOF 5600 mass spectrometer

5 μL of each sample, representing 50% of the sample, was directly loaded at 300 nL/min onto a New Objective PicoFrit column (15 cm × 0.075 mm I.D; Scientific Instrument Services, Ringoes, NJ) packed with Jupiter 5 μm C_18_ (Phenomenex, Torrance, CA) stationary phase. The peptides were eluted from the column by a gradient generated by an Agilent 1200 HPLC system (Agilent, Santa Clara, CA) equipped with a nano electrospray ion source coupled to a 5600 + Triple TOF mass spectrometer (Sciex, Concord, ON). A 65-min linear gradient of a 5–35% mixture of 0.1% formic acid injected at 300 nL/min was used to elute peptides. Data dependent acquisition mode was used in Analyst version 1.7 (Sciex) to acquire mass spectra. Full scan mass spectra (400 to 1250 m/z) were acquired and followed by collision-induced dissociation of the twenty most intense ions. A period of 20 seconds and a tolerance of 100 ppm were set for dynamic exclusion.

### Data dependent acquisition MS analysis

Data dependent acquisition MS analysis was performed essentially as per^56,67^ with minor modifications. Mass spectrometry data were stored, searched and analyzed using the ProHits laboratory information management system (LIMS) platform^68^. Within ProHits, AB SCIEX WIFF files were first converted to an MGF format using WIFF2MGF converter and to an mzML format using ProteoWizard (v3.0.4468) and the AB SCIEX MS Data Converter (V1.3 beta). The mzML and mzXML files were searched using Mascot (v2.3.02). The spectra were searched with the RefSeq database (version45, January 24th, 2011) acquired from NCBI against a total of 24,770 *T. thermophila* sequences. For Triple TOF files, the database parameters were set to search for tryptic cleavages, allowing up to two missed cleavage sites per peptide with a mass tolerance of 40 ppm for precursors with charges of 2+ to 4+ and a tolerance of +/−0.15 amu for fragment ions. Deamidated asparagine and glutamine and oxidized methionine were allowed as variable modifications. SAINTexpress version 3.61^43^ was used as a statistical tool to calculate the probability value of each potential protein-protein interaction from background contaminants using default parameters.

### MS data visualization and archiving

We used Cytoscape (V3.4.0)^69^ to generate protein interaction networks. Individual nodes were manually arranged based on the physiological condition examined. The annotation of the co-purifying partners was carried out using BLAST searches and SMART domain analysis (http://smart.embl-heidelberg.de/)^70^ of the predicted amino acid sequences which were acquired from the *Tetrahymena* genome database (www.ciliate.org). All MS files used in this study were deposited at MassIVE (http://massive.ucsd.edu), assigned the MassIVE accession numbers MSV000084066. Additional details (including Mass IVE accession numbers and FTP download links) can be found in Supplemental File S1.

### Identification and *in silico* analyses of HMG-proteins in *Tetrahymena*

We used the amino acid sequences of HMG-boxes present within *Tetrahymena* Mlh1 to search against the *Tetrahymena* genome database (www.ciliate.org). The proteins identified were analyzed by SMART domain analysis (http://smart.embl-heidelberg.de/)^70^ for the presence of HMGs. Phylogenetic analysis was carried out using the neighbor-joining method with 1000 bootstrap replicates. Amino acid sequences of the HMG-boxes from the identified proteins were used for the phylogeny reconstruction using MEGA7^71^. For gene expression analysis, microarray data (accession number GSE11300) was acquired (http://tfgd.ihb.ac.cn/) and the expression values were represented in the heatmap format. Z-scores were calculated across each row to examine the differential expression across different stages of the *Tetrahymena* life cycle. We used hierarchical clustering to examine the similarities in gene expression profiles. Mlh1 structural predictions were carried out using the I-TASSER server^72^. Hho1 secondary structure was predicted using PSIPRED^73^.

### Indirect immunofluorescence

*Tetrahymena* cells were grown and fixed during vegetative growth, after 24 hours starvation, and 2, 4, 6 and 7 hours post mixing cells of different mating types to perform indirect immunofluorescence. The IF analysis was performed essentially as previously described^56^. Briefly, cells were washed in 10 mM Tris-HCl, pH 7.7, and fixed in 4% paraformaldehyde. The cells were membrane-permeabilized with cold acetone for 20 minutes. Incubation with primary mouse anti-FLAG antibody (or anti-IgG (depicted as anti-FZZ)) (Sigma) was at a 1:500 dilution at 4 °C overnight in 1 × PBST. Cells were washed three times with 1 × PBS before incubation in secondary antibody fluorescein isothiocyanate-conjugated (FITC) goat anti-mouse (Pierce) for 1 hour at room temperature. For nuclear counterstaining 4,6-diamidino-2-phenylindole dihydrochloride (DAPI) was used. Immunofluorescence analysis was carried out using an Olympus, DP70 equipped with a fluorescent microscope (Reichert-Jung, POLYVER) at 100x magnification and no oil was used. Final image preparation was carried out using ImageJ (version 1.50i) software (https://imagej.nih.gov/ij/)^74^.

## Supplementary information

Supplementary information.

Supplementary information.
