# Supplementary material for: Nucleus-specific linker histones Hho1 and Mlh1 form distinct protein interactions during growth, starvation and development in Tetrahymena thermophila
Source: Sci Rep. 2020 Jan 13;10:168. doi: 10.1038/s41598-019-56867-0 (PMC6957481; doi:10.1038/s41598-019-56867-0)

Nucleus-specific linker histones Hho1 and Mlh1 form distinct protein interactions during growth, starvation and development in *Tetrahymena thermophila*

Syed Nabeel-Shah[1‡†], Kanwal Ashraf [2‡], Alejandro Saettone[1‡], Jyoti Garg[2‡], Joanna Derynck[1], Jean-Philippe Lambert[3], Ronald E Pearlman[2*] and Jeffrey Fillingham[1*]

1. Department of Chemistry and Biology, Ryerson University, 350 Victoria St., Toronto M5B 2K3, Canada.
2. Department of Biology, York University, 4700 Keele St., Toronto M3J 1P3, Canada.
3. Department of Molecular Medicine and Cancer Research Centre, Université Laval, Quebec, Canada; CHU de Québec Research Center, CHUL, 2705 Laurier Boulevard, Quebec, G1V 4G2, Canada.

[†] Present address: Donnelly Centre, University of Toronto, Toronto, M5S 3E1, Canada; Department of Molecular Genetics, University of Toronto, Toronto, M5S 1A8, Canada

[‡] equal contribution

[*] to whom correspondence should be addressed:

Jeffrey Fillingham, Department of Chemistry and Biology, Ryerson University, Toronto, M5B 2K3, Canada. Tel.: 416-979-5000 x 2123; E-mail: jeffrey.fillingham@ryerson.ca

Ronald E Pearlman, Department of Biology, York University, 4700 Keele St., Toronto M3J 1P3, Canada. Tel.: 416-736-5241; Email: ronp@yorku.ca

Supplemental data figures

Supplementary Figure S1: Microarray expression analysis of proteins that co-purify with Hho1 during vegetative growth, starvation and conjugation. The heatmap represents the hierarchical clustering of expression values for interacting proteins. Z-scores were calculated across the rows for each protein to examine its differential expression across growth, starvation and various developmental stages. L1-LH: the vegetative growth phase, S0-24: starvation for 24 hours, and C: conjugation where 0-18 denotes hours post mixing the different mating types. V, S and C in parenthesis after each prey protein name indicate vegetative, starvation and conjugation-specific interactions.

Supplementary Figure S2: *Tetrahymena* putative VIRMA protein. Domain analysis of *Tetrahymena* VIRMA protein in comparison to different species. The analysis was carried out using SMART (http://smart.embl-heidelberg.de/).

Supplementary Figure S1

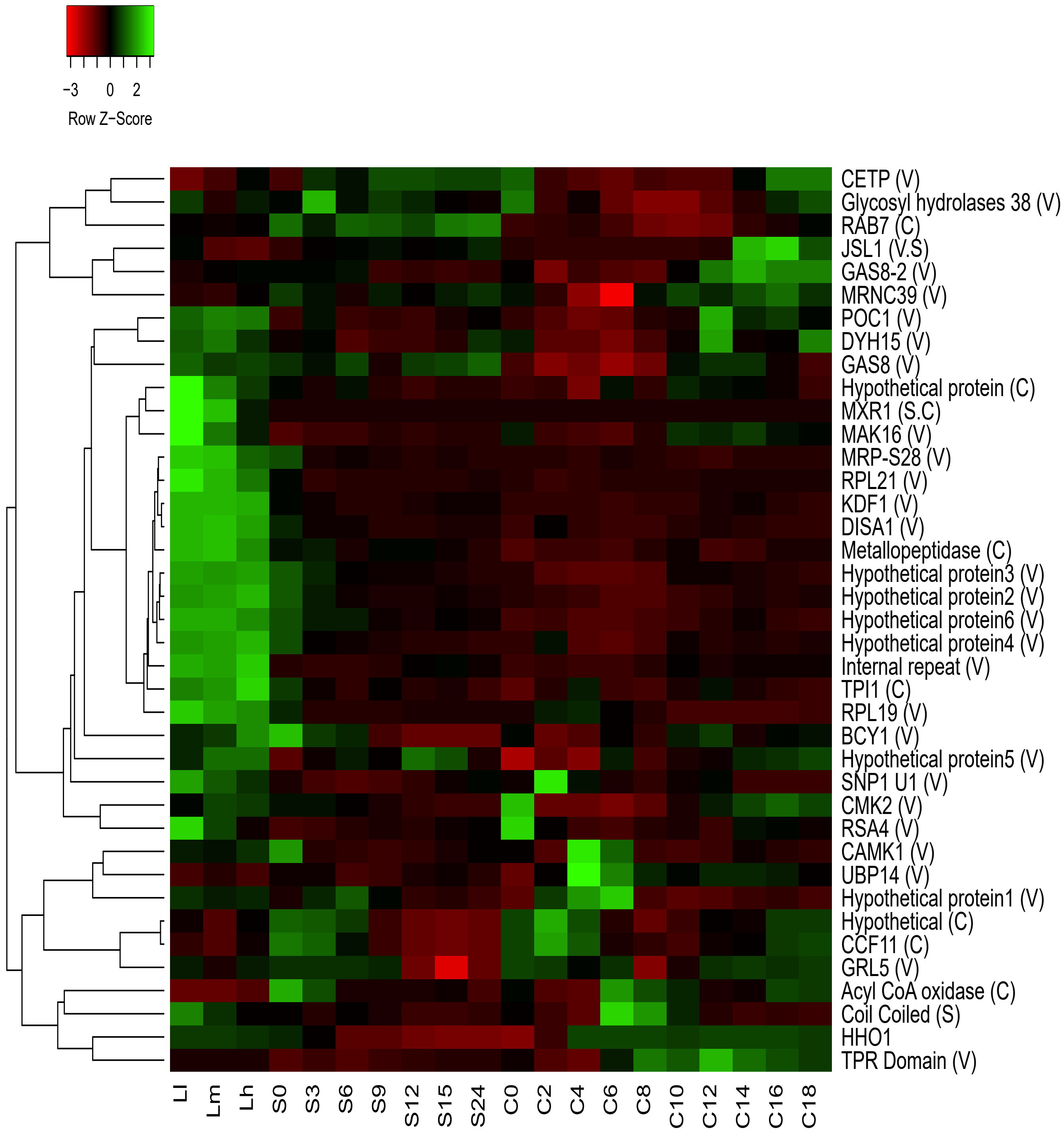

Supplementary Figure S2

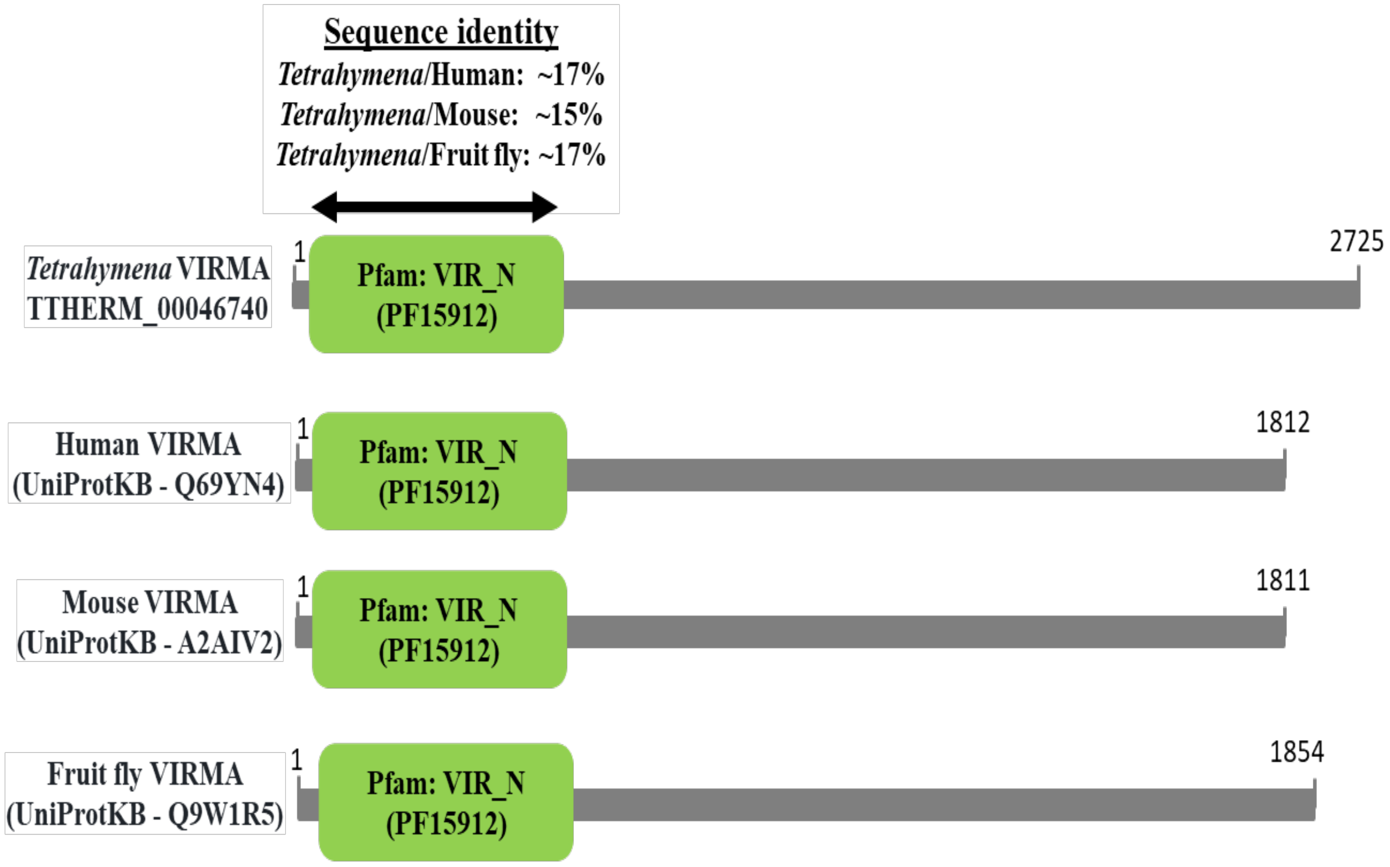

Supplement: Supplementary file 2 — Supplementary information. [file 41598_2019_56867_MOESM2_ESM.pdf]
